# Relational Memory Is Evident in Eye Movement Behavior despite the Use of Subliminal Testing Methods

**DOI:** 10.1371/journal.pone.0141677

**Published:** 2015-10-29

**Authors:** Allison E. Nickel, Katharina Henke, Deborah E. Hannula

**Affiliations:** 1 Department of Psychology, University of Wisconsin-Milwaukee, Milwaukee, Wisconsin, United States of America; 2 Department of Psychology, University of Bern, Bern, Switzerland; 3 Center for Cognition, Learning and Memory, University of Bern, Bern, Switzerland; University of Hyderabad, INDIA

## Abstract

While it is generally agreed that perception can occur without awareness, there continues to be debate about the type of representational content that is accessible when awareness is minimized or eliminated. Most investigations that have addressed this issue evaluate access to well-learned representations. Far fewer studies have evaluated whether or not associations encountered just once prior to testing might also be accessed and influence behavior. Here, eye movements were used to examine whether or not memory for studied relationships is evident following the presentation of subliminal cues. Participants assigned to experimental or control groups studied scene-face pairs and test trials evaluated implicit and explicit memory for these pairs. Each test trial began with a subliminal scene cue, followed by three visible studied faces. For experimental group participants, one face was the studied associate of the scene (implicit test); for controls none were a match. Subsequently, the display containing a match was presented to both groups, but now it was preceded by a visible scene cue (explicit test). Eye movements were recorded and recognition memory responses were made. Participants in the experimental group looked disproportionately at matching faces on implicit test trials and participants from both groups looked disproportionately at matching faces on explicit test trials, even when that face had not been successfully identified as the associate. Critically, implicit memory-based viewing effects seemed not to depend on residual awareness of subliminal scene cues, as subjective and objective measures indicated that scenes were successfully masked from view. The reported outcomes indicate that memory for studied relationships can be expressed in eye movement behavior without awareness.

## Introduction

Historically, questions about whether or not visual information can be processed and impact behavior in the absence of awareness have fostered considerable debate [1–3]. While there seems to be some agreement that subliminal perception can occur [4], questions remain about the nature or type of representational content that is accessible absent awareness [5]. Much of this debate has focused on the representational status of verbal materials that are presented below subjective or objective thresholds of awareness–specifically, whether or not semantic or conceptual representations can be recovered and influence behavior when words or digits are presented subliminally [6–8]. For example, it has been reported that participants can name, categorize, or otherwise respond to visible words more quickly when they are preceded by masked semantic associates than when they are preceded by unrelated words [9] (e.g. *doctor* preceded by the masked prime *nurse* as compared to *shoe*) and recent work indicates that incongruent relationships among scene elements (e.g., a man shaving with a fork rather than a razor) can be processed and impact subsequent responding to visible targets [10, 11].

It is noteworthy that in these examples, and others like them, associations are well-learned by virtue of the frequency with which they are encountered together in day to day experience. Far fewer investigations have examined whether or not memory representations for learned episodic relationships–e.g. the link between a place and a face–might also be recovered and impact behavior in the face of subliminal testing procedures. Therefore, the current investigation was designed to address questions about whether or not a subliminal cue might trigger implicit memory for scene-face relationships encountered just once prior to testing. This was addressed with two separate behavioral measures–namely, button-press responses and eye movements. Use of eye movement measures is an important contribution, as past investigations indicate that eye movements, which permit moment-by-moment evaluation of data, are sensitive to aspects of cognitive performance that are inaccessible to awareness and cannot be captured by discrete button-press or verbal responses [12]. In other words, eye movements may reveal effects of memory following subliminal scene cues when other types of behavioral responses fail to do so.

Several research investigations indicate that eye movement measures can be used to index remembered content [12], and collectively, results from these studies are consistent with the view that eye movements may be an especially sensitive index of memory. For example, recent work has shown that effects of memory on eye movement behavior are evident shortly after display onset [13–17], occur even when memory-based viewing is counterproductive based on task requirements [16, 18], and that eye-movement-based memory effects can be documented in the absence of conscious awareness [19–25]. Related to this last point, it has been reported that participants prioritize (i.e. look disproportionately at) regions of scenes that have been manipulated at test even when they cannot explicitly describe or recognize the changes that have been made [24, 25]. This pattern of results is provocative and suggests that awareness is not a prerequisite for memory-based viewing. However, it is also the case that this outcome has not always been successfully replicated [26, 27], and therefore, may not provide iron clad evidence in support of the view that eye-movement-based memory effects can be documented without concomitant awareness of retrieved content. More generally, skeptics might argue that the methods used to assess awareness in these experiments were insufficiently sensitive. This is because participants have typically been asked to report whether or not scenes were manipulated, describing the change if one was present. Under these circumstances, participants may opt to make conservative responses, indicating “no change” when they suspect things are different but lack confidence in the accuracy of their memories [28].

The use of subliminal memory cues in the current study meant that we could examine whether or not eye movements are sensitive to memory for studied relationships when participants do not “see”, and therefore remain unaware of, the retrieval cues. Furthermore, administration of both subjective and objective assessments of awareness subsequent to completion of the experiment proper meant that we could confirm stimulus visibility had been successfully controlled. Finally, the application of a regression approach developed by Greenwald and colleagues [29] meant that we could evaluate the reliability of any observed implicit effects when performance on the objective test of awareness was regressed to zero (see also [30] for a brief summary of this method). These methodological choices were made to address some of the criticisms that have been levied against past reports.

Consistent with the feasibility of the subliminal masking approach, a collection of studies conducted by Henke and colleagues indicate that subliminally encoded content can sometimes influence the speed and accuracy of judgments that are made when supraliminal test displays are presented [31]. For example, results from two investigations have shown that when participants are asked to guess the occupational category of a person seen in a picture (i.e. “artist” or “academic”), their responses are faster when they make guesses that are consistent with a previous subliminal study exposure [31–32]. If, for example, a face had been paired with the occupation “pianist” during subliminal encoding, then responses would be faster if participants guessed “artist” when that face was re-presented visibly, than if they guessed “academic”. This outcome suggests that the face-occupation association had been learned and could influence the speed of subsequent decision making. More recently, work from the same group has indicated that participants can encode shared associations across subliminally presented word pairs [33] (see also [34–36] for subliminal learning of associative analogies). For instance, if the word pairs *bicycle-rain* and *rain-pen* are presented subliminally during an encoding phase, and then the pair *bicycle-pen*, related by virtue of the common associate *rain*, is presented for supraliminal inspection, participants are more likely to indicate that these pre-experimentally unrelated words are a “good fit” than if they did not share a common associate during subliminal encoding. This outcome suggests that participants have processed each pair, and that the connection across pairs has been inferred and encoded despite an absence of awareness. Collectively, these experiments indicate that participants can learn novel face-occupation pairs and word pairs despite the fact that they were presented in the context of a subliminal masking sequence that rendered them invisible during encoding, and that the resulting memory representations exhibit properties that characterize episodic memory (e.g., rapid encoding and representational flexibility) [37].

Like the studies described briefly above, the current investigation addresses questions about whether or not memory for arbitrarily paired items can be retrieved in the absence of awareness. In contrast to past work, however, the encoding experience here is supraliminal and a memory cue, meant to trigger retrieval of relational memory representations at test, is subliminal. In this case, memory expression requires not only that the subliminal cue is processed, but that it triggers pattern completion processes mediated by the hippocampus [38] that permit access to an associate encountered just once in combination with the cue during supraliminal encoding. Another departure from past work concerns the use of eye movement measures to tap expressions of memory, an approach that is integrated here with standard indices of response time and accuracy.

Participants in this experiment were asked to commit several (visible) scene-face pairs to memory. During a corresponding test phase, each trial began with the subliminal presentation of a studied scene cue (i.e., a scene cue that was masked from awareness). Following the subliminal scene cue, a visible 3-face display was presented and eye movements were recorded. For participants assigned to an experimental group, one of the faces was the studied associate of the scene cue; for control group participants, the associate was not present in the display. Regardless of group assignment, participants were instructed to select the face they felt was likely to be the studied associate of an upcoming visible scene cue, and they were told that this task was a measure of foresight. Use of this first test display meant that we could evaluate eye-movement-based and behavioral measures of implicit memory. Subsequent to the implicit test, the same 3-face display was presented again, but now it was preceded by the visible scene. In this case, the studied associate was present regardless of group assignment and participants were instructed to select that face from the alternatives while their eye movements were being recorded. Viewing patterns and behavioral responses to this display were used to assess explicit memory for the studied scene-face relationships.

As reported in past work [13, 15, 16, 39, 40, 41], we expected that eye movements would be allocated disproportionately to faces that had been paired with specific scene contexts, but here these viewing effects were expected even when the scene cues, meant to trigger memory for studied relationships, were presented subliminally. Specific predictions about the time-course of memory-based viewing to implicit and explicit test displays are outlined below (in the methods section), following a detailed description of the procedures that were used to analyze the eye movement data. Consistent with results reported by Henke and colleagues [32, 36], it was predicted that participants assigned to the experimental group would respond more quickly when associates were identified correctly (vs. not) following subliminal scene cues, which would indicate that the speed of decision making was influenced by retrieved relationships. It was also possible that presentation of the subliminal scene cue would drive response selection accuracy among experimental group participants–i.e., that the associate would be selected more often than chance. On the other hand, and as described in the discussion section, button-press responses may prove insensitive to memory in the face of subliminal stimulus presentations; for that reason evaluation of the eye movement record was considered particularly important. Ultimately, any evidence for memory in button-press responses and/or the eye movement record would complement and extend past work using subliminal encoding procedures, and would provide additional evidence in support of the view that episodic memory representations can be accessed and expressed even in the absence of awareness.

## Methods

### Participants

Forty students from the University of Wisconsin–Milwaukee completed this experiment and were compensated with course credit. After providing written informed consent in a manner approved by the Institutional Review Board at UWM, participants were randomly assigned to either an experimental group (N = 20) or a control group (N = 20). Data obtained from one participant assigned to the experimental group were dropped from reported analyses because explicit recognition performance was more than two standard deviations below the group mean and not better than chance. Therefore, all of the reported analyses were based on data obtained from 39 participants, 19 assigned to the experimental group and 20 assigned to the control group. Seven additional participants were dismissed before testing commenced because successful calibration could not be achieved. All of the procedures for this experiment were approved by the local Institutional Review Board at UWM.

### Materials

One hundred sixty-eight gray-scale scenes were selected for the current study based on the outcome of a norming experiment. Half of these pictures were indoor scenes (e.g., dentist office) and the remainders were outdoor scenes (e.g., Millennium Park). The norming experiment was conducted to ensure that each scene could be successfully masked from view when it was presented subliminally in the experiment proper.

**Fig 1:**
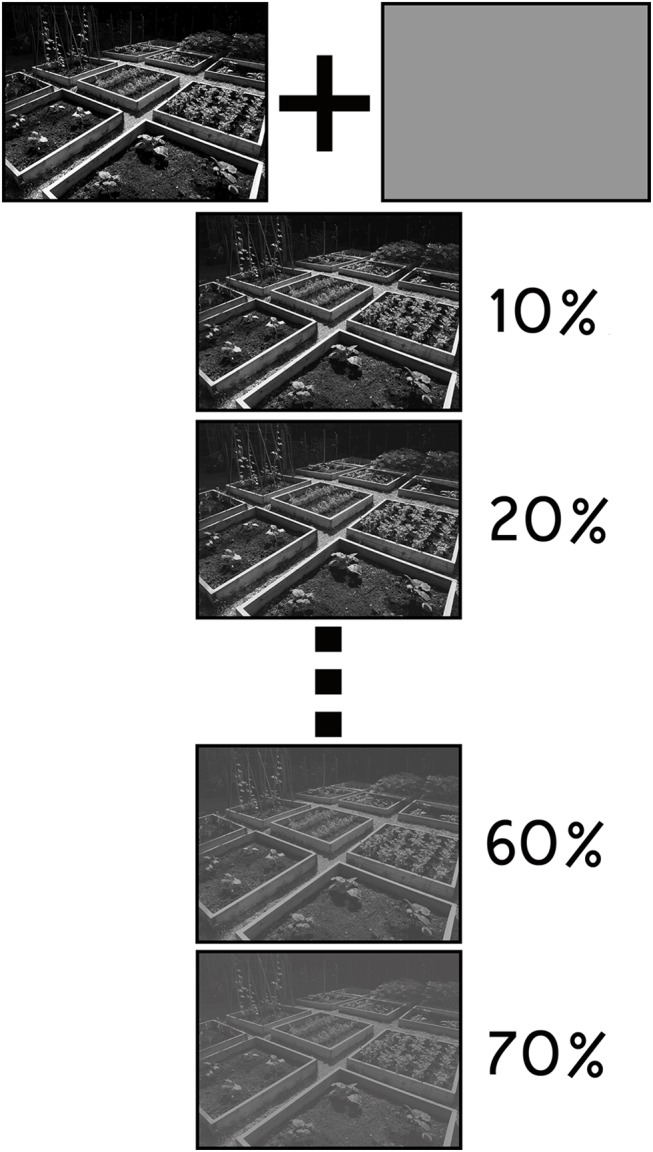
Scene Degradation. Illustration of the approach that was used to systematically degrade scenes that were presented in the subliminal masking sequences. A gray layer (R: 115, B: 113, G: 113) was superimposed on top of each scene. The opacity of this layer was then systematically increased to produce a set of nine additional scene exemplars that were used in a norming experiment. Scenes carried forward for use in the experiment proper were selected based on the outcome of the norming investigation.

For norming purposes, each scene was systematically degraded in a process that yielded a set of nine additional exemplars. To produce each set, a gray layer (R: 115, B: 113, G: 113) was superimposed on top of the original scene and the opacity of this layer was repeatedly increased by 10% so that the visibility of the picture underneath was systematically reduced (see Fig 1). Resulting images were embedded in a subliminal masking sequence [31] to determine which version of each picture would be carried forward for use in the primary investigation. Among the subset of scenes that was selected, the maximum level of required degradation was 70%. Ultimately, 96 scenes in the final set were successfully masked from view during norming without any degradation. Remaining scenes had superimposed gray-scale layers with opacities set to 10 (15 pictures), 20 (10 pictures), 30 (9 pictures), 40 (8 pictures), 50 (11 pictures), 60 (14 pictures), or 70 (5 pictures) percent, respectively (see S1 Supplementary Materials for more information about the norming procedure).

In addition to the final set of scenes, 168 gray-scale faces (half male, remainder female) were selected from an established faces database [16] and forty-two black and white visual noise images were used as masks when scenes were presented subliminally [32].

The total screen resolution was set to 800x600 pixels. Scenes and masks were presented centrally, and were sized to 600x450 pixels; faces were 210 pixels square and were superimposed on a 225x225 pixel gray background. When scenes were presented side by side during the 2-alternative forced-choice awareness assessment they were sized to 300x225 pixels.

### Apparatus

Eye position was recorded every 17ms (i.e., at a sampling rate of 60 Hz) with an Applied Science Laboratories D6 remote eye tracker (Applied Science Laboratories, Bedford, MA). This eye tracking system illuminates the eye with infrared light and records changes in the angle between the pupil and the corneal reflection as the eyes move. Head movement in all three dimensions was recorded using a head tracking system and head position was integrated with eye position to permit reliable acquisition of data that reflects gaze coordinates. Event timing was controlled with Presentation software (Neurobehavioral Systems, Berkeley, CA) and stimuli were viewed on an ASUS LCD monitor with a 60 Hz refresh rate.

### Design and Procedure

After providing informed consent, participants were seated at a distance approximately 25 inches from the computer monitor and task instructions were provided. Following a brief practice session, participants had an opportunity to ask any remaining questions and then eye position was calibrated using a 3 x 3 spatial array, a process that was repeated prior to each experimental block. As in previous studies [13–16], elements in the array (i.e. the letters A through I) were positioned in three equally spaced rows and columns across the screen, covering the bounds of our stimulus displays. Participants were asked to fixate each letter in turn, eye position was recorded, and then the alignment of fixations with calibration points was checked by the experimenter. If fixations did not fall on the calibration points, the process was repeated.

**Fig 2:**
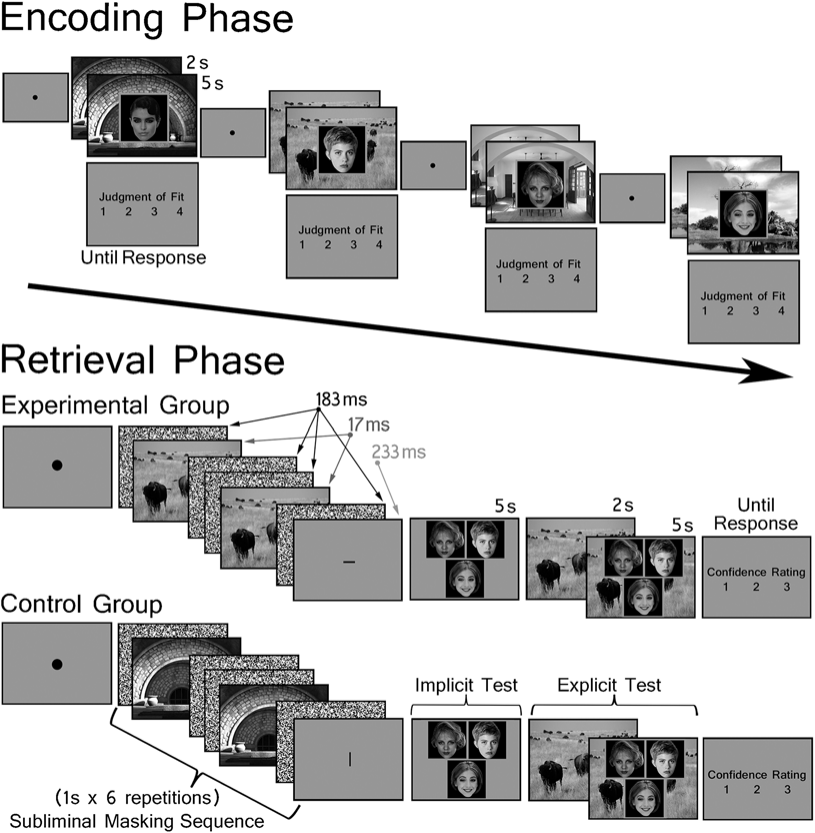
Encoding and Retrieval Trials. *(top)* Scenes were presented alone for 2 seconds at the start of each encoding trial. A face was then superimposed on the scene for 5 seconds and participants were instructed to indicate whether the pair was a good fit using a scale from 1–4. There was no time limit imposed on the judgment. *(bottom)* Each test trial began with the subliminal presentation of a studied scene. The scene was embedded in a masking sequence and participants were instructed to perform an attention task–making a button press when a visible fixation cross was replaced with a vertical or horizontal line segment. The masking sequence shown here was repeated six times, which meant that the scene was presented twelve times in 6 seconds. Following subliminal presentation of the scene, a visible three-face display was presented for 5 seconds and was used to test memory implicitly. At the end of the trial, a visible scene cue (2 seconds) preceded presentation of the same 3-face test display (5 seconds), which was used to test memory explicitly. Controls were yoked to experimental participants so that all of the trial events were the same with the exception of the subliminal scene cue, for which there was not an associate in the corresponding implicit test display.

Individual encoding blocks were followed by corresponding test blocks; altogether, three encoding-test block sequences were completed by each participant. During each encoding block, participants were presented with 56 trial-unique scene-face pairs and were instructed to commit each pair to memory. Individual trials were experimenter-initiated contingent upon participants fixating a centrally-located black circle. When the trial advanced, a scene was presented for 2 seconds, and then a face was superimposed on top of the scene for 5 seconds. Following the presentation of each pair, participants made a button press to indicate whether or not they felt there was a good fit between the person and the place depicted by the scene (1 = Terrible Fit, 2 = Poor Fit, 3 = Good Fit, 4 = Excellent Fit); a time limit was not applied to this judgment, which was meant to encourage deep encoding of each pair (see Fig 2).

Fourteen trials were presented in each test block and were experimenter-initiated contingent upon participants fixating a centrally-located black circle. Each trial began with the subliminal presentation of a studied scene (described below) and was followed by the presentation of a visible 3-face display, which remained in view for 5 seconds and was used to evaluate *implicit* retrieval of the studied scene-face relationship. All three faces in this display had been seen during the corresponding encoding block and participants were asked to select the face that they *expected* would be the associate of an upcoming visible scene cue. Participants were told that this was a measure of foresight, a cover story that permitted us to obtain behavioral responses to 3-face displays preceded by subliminal scene cues (i.e., a behavioral measure of implicit memory). Immediately thereafter, a visible scene cue was presented for 2 seconds. Finally, the same 3-face display was re-presented, superimposed on top of the scene for 5 seconds; this display was used to evaluate *explicit* retrieval of the scene-face relationship. At this point, participants were instructed to indicate which face had been paired with the scene during the encoding phase (i.e., a behavioral measure of explicit memory) by making a button press. Finally, participants were asked to indicate how confident they were in the accuracy of their explicit recognition response (1 = high confidence, 2 = low confidence, 3 = guess; see Fig 2).

Participants were not told about the presence of the scene in the masking sequence; instead, they were instructed to perform an attention task, which was used to minimize any potential differences in the distribution of attention across the visual display. Successful performance of this task required participants to direct attention to a centrally-located fixation cross, and to make a button press when the cross was replaced with a horizontally- or vertically-oriented line segment. To conceal the purpose of the masking sequence, participants were told that the task was simply meant to index attention. The masking technique developed by Henke and colleagues [32] was used to present the scene cue subliminally at the start of each test trial. Using this technique, each scene cue was presented twelve times in 6 seconds, flanked by visual noise masks. Presentation durations were 17ms for the scene (S), 183ms for masks (M), and 233ms for the fixation crosses/line segments (F). The subliminal presentation of one scene was given in the following sequence F-M-S-M-M-S-M-F-M-S-M-M-S-M-F-M-S-M-M-S-M-F-M-S-M-M-S-M-F-M-S-M-M-S-M-F-M-S-M-M-S-M and the fixation cross (F) was replaced randomly with a horizontal or vertical line segment once per trial.

As indicated above, participants were randomly assigned to an experimental group or a control group prior to testing. For participants in the experimental group, the *subliminal* scene cue was the studied associate of one of the faces in the corresponding 3-face display. Participants assigned to the control group were presented with the same 3-face displays (i.e. each control group participant was yoked to an experimental group participant), but the subliminal cue was a different studied scene for which there was not a match. Yoked participants assigned to the experimental and control groups were presented with the same *visible* scene cue, which always had a match (i.e. a face with which it had been studied) in the 3-face display presented at the end of the test trial (see Fig 2).

The above scheme meant that participants assigned to the experimental group were presented with 14 scene cues in each test block–i.e. the same scene was presented subliminally and supraliminally, and this scene was the studied associate of one of the faces in each of the corresponding 3-face displays. In contrast, participants assigned to the control group were presented with 28 scene cues in each test block–i.e. 14 subliminal scenes for which there was not a match in the corresponding 3-face display and 14 *different* supraliminal scenes for which there was a match. Across trials, the *critical face* (i.e. the studied associate of subliminal scene cues for the experimental group, and the same face absent the association for the control group) appeared equally often in every spatial location (i.e., left, right, or bottom). Altogether, participants completed 42 test trials in this experiment.

Upon completion of the experiment proper, participants provided responses to a short post-test questionnaire similar to one that has been used previously by Henke and colleagues [32]. This questionnaire was used to determine whether or not participants had suspicions about the purpose of the masking sequence or had noticed any regularity in the visual noise images while they were performing the attention task. After responses to these initial questions were obtained, the purpose of the masking sequence was revealed and participants answered several additional questions (e.g., Now that you know about the presence of the scene, do you feel that you may have been aware of it on some of the trials?). Responses to these questions provided a subjective measure of scene perception and recognition.

**Fig 3:**
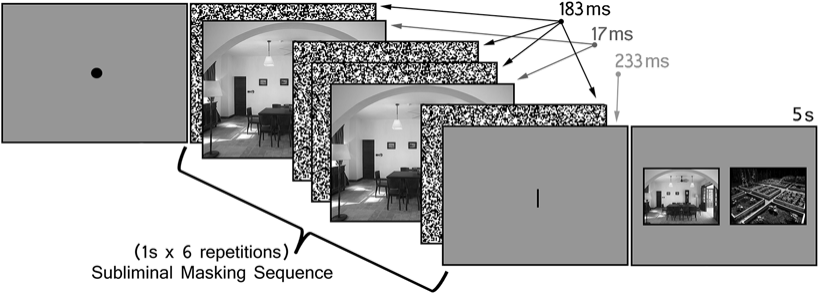
Objective Test of Scene Awareness. Individual trials began with the subliminal presentation of a studied scene. Participants were told that scenes were embedded in the masking sequences and were asked to select that scene from two alternatives presented for 5 seconds at the end of the trial.

Subsequent to questionnaire administration, all of the participants completed a more objective test of awareness. Prior to administration of this test, participants were told that a masking sequence, identical to the one used previously, would be presented, but now they were aware that a scene was embedded among the visual noise images. With this in mind, they were asked to identify that scene from two alternatives following presentation of the visual noise display. As in the experiment proper, participants performed the attention task while the masking sequence was in view (see Fig 3).

The forced-choice awareness test was subdivided into three blocks of 14 trials (42 trials altogether, as in the primary experiment). Scenes from pairs that had been encoded, but had not appeared in the test phase, served as the materials for this phase of the experiment. Use of encoded scenes was important because it is possible that pictures embedded among visual noise masks are more easily perceived and/or identified when they have been seen previously. All of the scenes were presented in reverse order relative to when they had been encoded (scenes encoded more recently were presented in the first block of the awareness test and those encoded more remotely were presented in the final block of the awareness test). This approach meant that we could examine whether or not successful recognition of subliminally presented scenes was affected by the amount of time that had passed since encoding. Each two-alternative forced-choice display remained in view for 5 seconds and participants made a button press to identify the scene that they felt had been presented in the corresponding masking sequence. Across trials, the masked “target” scene appeared equally often to the left and right of fixation.

### Counterbalancing

For counterbalancing purposes, individual scenes were randomly assigned to one of 12 lists (14 scenes per list), each with equal numbers of indoor and outdoor exemplars. Faces were also assigned to one of 12 lists (14 faces per list), each with an equal number of male and female exemplars. For each participant, a given list of scenes was then paired with a given list of faces. Individual scenes and faces from corresponding lists were presented as pairs during the encoding phase. One-quarter of the encoded scenes and three quarters of the encoded faces were used to construct retrieval trials for the experimental group. Half of the encoded scenes and three quarters of the encoded faces were used to construct retrieval trials for the control group. The remaining scenes, those that were not presented in retrieval trials for either group, were used in the objective awareness test. Lists of scenes and faces rotated across experimental conditions and blocks, and faces from each list rotated across “face types” in the 3-face displays. Because there were two foil faces for every critical face in a given display, this meant that each individual face exemplar was presented more often as a foil than as a critical face across participants. This is important because it meant that perceptual factors (e.g. the physical appearance of a particular face) could not confound any of the observed memory effects. In other words, any bias toward a small subset of faces based on perceptual characteristics would work against us because, across participants, each face was overrepresented as a foil.

### Eye Movement Analyses

As in past work (see [12] for review), fixations were defined by successively recorded samples with gaze position changes less than 1° of visual angle that, when combined, had a minimum duration of 100ms. Individual fixations were then assigned to a given region of interest–i.e. one of the three faces (with a bounding box that incorporated the black background on which faces were superimposed) or the remainder of the screen. Off screen fixations were eliminated from further analysis.

Two classes of eye movement measures were used to evaluate the impact of memory on patterns of viewing. *Global measures* were calculated based on proportion of total viewing time directed to specific faces of interest collapsed across the duration of the entire test trial, whereas *time-course measures*, which provide more precise information about how viewing unfolds over the course of a test trial, were calculated after the data had been subdivided into consecutive 250ms time bins. A time-course analysis was performed because past work has shown that global measures do not always reveal effects of memory [16]. For the time-course analysis, data from the first 2 seconds following 3-face display onset were considered. Analyses were limited to early viewing because past work shows that memory-based viewing effects are most robust shortly after test display onset [13, 14, 16, 18] and the 2000ms cutoff has been used in past work with the same basic paradigm [16].

Eye position was frequently unreliable immediately after the subliminal masking sequence was presented because pupil size was affected by the flashing checkerboard display. Therefore, data from the first time bin (0–250ms) were dropped from all of the reported statistical tests. Displays for which eye movement data were lost or unreliable (i.e. total viewing time less than 65% the display duration) were also dropped from reported analyses [14]. Using this approach, average elimination was 7 or 8 trials per subject for implicit and explicit test displays, respectively. Participants with an insufficient number of remaining trials in a bin of interest (i.e. fewer than 4) [14] were eliminated from reported contrasts. When this occurs, it is noted in the text. In the paragraphs that follow, more specific information about how data were analyzed for the implicit and explicit test displays is provided along with corresponding predictions.

Implicit Test (following subliminal scene cues): Individual implicit test displays were subdivided into two types based on button press responses that were made by the participants: 1) trials characterized by selection of the critical face, and 2) trials characterized by selection of a foil face. When a foil was selected, the remaining two faces in the display (i.e. the critical face and the second foil) were also used in reported analyses. This meant that there were four “faces of interest”: 1) selected critical faces, selected foils, critical faces not selected, and foils not selected. Based on past work with this paradigm [16], we know that viewing is affected by two factors: 1) the act of selection, and 2) memory for scene-face relationships. Selected faces tend to be prioritized over faces that are not selected, and associates tend to be prioritized over foils.

There are two ways to document memory-based viewing effects after equating for selection history–namely, greater viewing of selected associates than selected foils, and greater viewing of associates that were not selected than foils that were not selected. Use of all three faces from a given test display (i.e. when a foil face has been selected) in reported comparisons might appear problematic because viewing directed to one face necessarily detracts from the remaining two. Critically though, it is also the case that if memory has no impact on eye movement behavior, residual viewing (regardless of time spent on the selected foil) should be equally distributed among the other two faces in the display (i.e. the associate and the remaining foil); in the absence of a memory-based contribution, there is no reason to prioritize the associate.

Comparisons among face types were performed using global measures (collapsed across the entire test trial) and time-course measures as described in the preceding section. Relative to corresponding foils, it was predicted that experimental group participants would show disproportionate viewing of critical faces that were *not selected* within 1000–2000ms of 3-face display onset [15], and that disproportionate viewing of selected critical faces would occur earlier (e.g., within 500–750ms of display onset; 14–16). Controls were expected to show effects of selection, but were not expected to prioritize critical faces over corresponding foils because, for this group, the critical face had not been paired with the subliminal scene cue during encoding. In addition, it was considered unlikely that effects of memory would be evident in global viewing measures, as these are not sensitive to transient effects of memory [16].

Explicit Test (following visible scene cues): The same basic approach was used to examine viewing directed to explicit test displays, but now scene cues were visible, and critical faces were associates of the scene cues for both groups of participants. As above, individual trials were subdivided into two types–those characterized by correct identification of the critical face (i.e. the associate) and those characterized by incorrect identification of a foil as the associate. Effects of selection and memory were evaluated as described above. Consistent with past work [16, 41], disproportionate viewing of correctly identified associates (relative to foils selected in error) was expected shortly after display onset (i.e. within 500–750ms of 3-face display presentation) for both groups. Disproportionate viewing of non-selected associates was expected to emerge later in time, and would suggest an influence of memory in the absence of conscious awareness. However, this evidence might be considered less compelling than results from the implicit test because the possibility remains that participants simply pressed the wrong button, or realized that their response was incorrect, but failed to rectify the error. Finally, in the presence of visible scene cues and explicit retrieval instructions, memory-based viewing effects were expected to be longer lasting, and might therefore be evident in global measures as well.

### Statistical Contrasts

Mauchly’s test of sphericity was calculated for all of the reported ANOVAs with more than one degree of freedom in the numerator. In the event that sphericity was violated, Greenhouse-Geisser adjusted degrees of freedom, p-values, and corresponding epsilons (G-G ε) were reported. Post-hoc statistical tests were Bonferroni corrected for multiple comparisons, and Cohen’s d was calculated as a measure of effect size using means and standard deviations from each group.

## Results

### Behavioral (Button-Press) Performance

#### Attention Task

Results indicated that participants complied with attention task instructions, as they failed to make responses on just 5.19% (SD = 12.24) of the test trials. For the remaining trials, successful detection/identification of horizontal or vertical line segments embedded in the subliminal masking sequence was high and well-matched across groups (Experimental Group: 82.61% correct, SD = 16.60; Control Group: 83.78% correct, SD = 21.58; t(37) = .08, p = .94; Cohen’s d = .03). This outcome indicates that the allocation of attention to subliminal scene cues during test trials was comparable among experimental and control group participants.

#### Implicit Memory for Scene-Face Relationships

Based on previous reports [30], memory-based differences in decision making and/or response time were predicted for experimental group participants. Specifically, it was expected that following presentation of the subliminal scene cue these participants might select critical faces more often than would be expected by chance, or that their responses might be made more quickly when critical faces happened to have been selected versus not. The same outcomes were not expected for controls, as there was not a learned relationship between subliminal scene cues and critical faces embedded in 3-face displays for this group (see Fig 2).

Initial evaluation of the behavioral data indicated that participants failed to make responses on 3.11% (SD = 5.81) of the trials. After discarding this small subset of the data, it was determined that neither group selected critical faces more often than would be expected by chance (Experimental Group: 34.77% selection, SD = 5.99%, t(18) = 1.29, p = .11; Control Group: 34.83% selection, SD = 7.55, t(19) = 1.09, p = .14), and there were no differences in the rate with which critical faces were selected across groups (t(37) = .01, p = .99; Cohen’s d = 0). In sum, there was no evidence for any influence of scene-face memory on experimental group performance following presentation of the subliminal scene cue.

To determine whether or not there were response time differences between groups when the critical face happened to have been selected (vs. not), a between-groups repeated-measures ANOVA was calculated with the factors group (experimental vs. control) and trial type (critical face selected vs. critical face not selected). In contrast to our predictions, the amount of time required to select a face was not influenced by whether or not that face happened to have been the studied associated of the subliminal scene cue. Results indicated that response times were numerically longer for controls than for participants assigned to the experimental group, but these differences were not statistically significant (F(1,37) = 2.65, p = .12). More important for our purposes, there were no response time differences as a function of trial type (F(1,37) = .03, p = .87), nor was there a statistically significant group by trial type interaction (F(1,37) = .59, p = .45; see Table 1). Therefore, results reported in past work [31, 32] were not replicated in these initial contrasts.

**Table 1:** Response Time (ms) Associated with Face Selection Following 3-Face Display Onset.

	Experimental Group		Control Group	
	*Critical Face Selected*	*Foil Face Selected*	*Critical Face Selected*	*Foil Face Selected*
**Implicit Test**	2466.03 (731.77)	2490.88 (810.75)	2838.81 (618.82)	2799.86 (468.84)
**Explicit Test**	2113.99 (484.42)	2900.26 (686.19)	2232.04 (405.64)	3140.85 (491.84)
**Back-Sorted**	2440.37 (679.20)	2488.90 (801.07)	2812.90 (604.22)	2760.10 (481.19)

Standard deviations of the mean are in parentheses.

#### Explicit Memory for Scene-Face Relationships

Explicit memory for scene-face relationships was tested with 3-face displays presented subsequent to visible scene cues. In this case, the scene cue was the studied associate of the critical face for participants assigned to both groups. Here, participants were instructed to identify that face from among the three alternatives and it was predicted that there would be no differences between groups in accuracy or response time.

Participants failed to make responses on 3.85% (SD = 6.22) of the explicit test trials, and evaluation of explicit recognition performance on the remaining trials confirmed our predictions. Recognition memory accuracy was reliably above chance for both groups (Experimental Group: 75.92% correct, SD = 16.14, t(18) = 11.59, p<. 001; Control Group: 83.33% correct, SD = 10.81, t(19) = 21.20, p<. 001), and there was not a between-groups difference in performance (t(37) = 1.66, p = .11; Cohen’s d = .52).

A repeated-measures ANOVA with the factors group (experimental vs. control) and accuracy (associate correctly identified vs. associate not identified) indicated that response times were also well-matched across groups (F(1,28) = 0.89, p = .35), and that group assignment did not interact with trial type (F(1,28) = .75, p = .39). There was, however, a main effect of recognition accuracy–responses were made more quickly when the associate was correctly identified than when one of the foil faces was selected in error (F(1,28) = 109.93, p < .001; see Table 1). Note that 9 participants were excluded from this analysis because they failed to meet the 4 trial bin size criterion for incorrect responses. However, when the bin size requirement was omitted, and just one participant was eliminated because no incorrect responses were made, the statistical outcomes remained the same. There were no group differences in response time (F(1,36) = .31, p = .58), nor was there a statistically reliable interaction (F(1,36) = .19, p = .66), but participants made faster responses when the associate was correctly identified (F(1,36) = 98.25, p < .001).

#### Subsequent Memory Analyses

To account for the possibility that participants may have failed to encode some of the studied pairs, implicit behavioral data were back-sorted as a function of response accuracy on the explicit recognition test. Here, data analysis was limited to the subset of implicit trials for which participants successfully identified the associate on the corresponding test of explicit memory. This approach was used to determine whether or not implicit memory effects might be evident for the subset of scene-face pairs that had been learned during encoding.

When analyses were limited to successfully recognized pairs, participants assigned to the experimental group selected critical faces more often than would be expected by chance from the initial 3-face test display (40.15% selection, SD = 13.75%, t(18) = 2.50, p = .02). This result suggests an influence of memory for scene-face pairs on the selection process following presentation of subliminal scene cues. However, the same effect was marginal among control group participants (36.25% selection, SD = 8.52, t(19) = 1.91, p = .07), and a direct comparison indicated that the frequency with which critical faces were selected was not reliably different across groups (t(37) = 1.07, p = .29; Cohen’s d = .33). Because the presence of a trend toward greater selection of critical faces among controls was unanticipated, exploratory analyses were conducted to determine whether observed outcomes might be related to some sort of behavioral response bias. As is reported in the S1 Supplementary Materials, perceptual and location-based factors were ruled out, but results did reveal a subtle tendency for participants in both groups to select the same face from corresponding implicit and explicit test displays. This observation is notable because when analyses are back-sorted based on explicit recognition accuracy, and there is a bias towards repeated choices, correct implicit recognition responses may be artificially inflated. Consequently, it cannot be said with certainty that the above-chance selection of critical faces among experimental group participants reflects memory. As above, results from a repeated measures ANOVA with the factors group (experimental vs. control) and trial type (critical face selected, critical face not selected) did not provide evidence in support of predicted response time differences (all F’s(1,36)≤2.27, p’s≥.14; see Table 1). One participant was eliminated from this response time analysis because there were fewer than four incorrect trials remaining in the back-sorted data set.

### Eye Movements

#### Implicit Memory for Scene-Face Relationships

**Fig 4:**
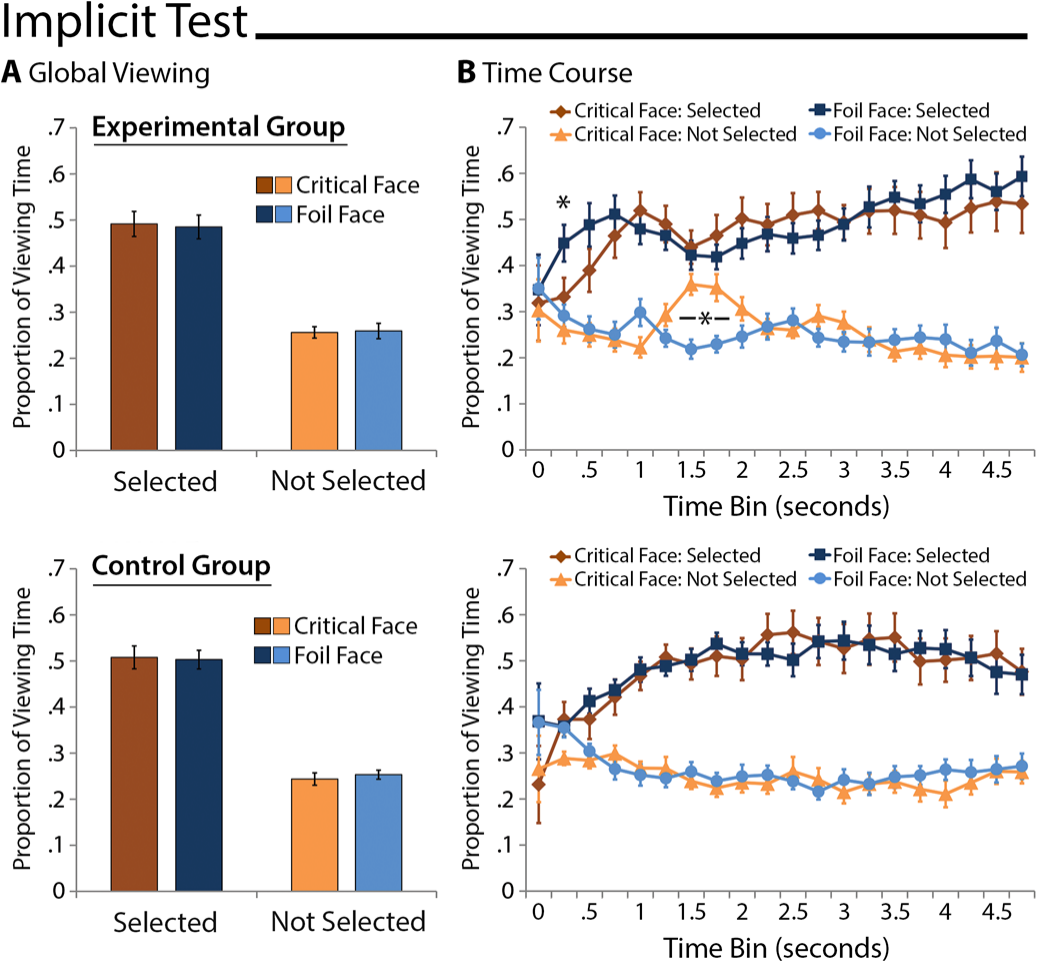
Implicit Memory-Based Viewing Effects. A) Proportion of total viewing time directed to faces of interest collapsed across the duration of the entire test trial for the experimental group (top) and the control group (bottom). More time was spent looking at faces that happened to have been selected, but there were no effects of memory in viewing patterns. B) Proportion of total viewing time direct to faces of interest broken down into 250ms time bins starting with 3-face display onset. Faces that happened to have been selected were prioritized, but effects of memory on the viewing behavior of experimental group participants (top) are also evident; the same effects are absent from control data (bottom). Error bars indicate standard errors of the mean.

*Global Measures*: There were no differences in the proportion of total viewing time directed to critical faces (selected or not) relative to corresponding matched comparison faces for either group (Experimental Group: t’s(18) ≤ .31, p’s ≥ .76; Control Group: t’s(19) ≤ .80, p’s ≥ .44). A between-groups repeated measures ANOVA with the factors face type (critical face, comparison foil face) and selection history (selected, not selected) indicated that more time was spent viewing faces that happened to have been selected vs. not (F(1,37) = 101.24, p < .001), but none of the remaining main effects or interactions were statistically reliable (all F’s ≤ .52, p’s ≥ .48; see Fig 4A). This outcome was not unexpected, as previous work indicates that memory-based viewing effects may be especially robust soon after test display onset, and can be obscured when data are collapsed across the entire duration of a test display (cf. Hannula et al., 2007). Time-course measures, described next, were used to determine whether or not there were differences early in viewing that distinguished studied associates from corresponding matched comparison faces.

*Time-Course Measures*: A between-groups repeated-measures ANOVA with the factors group (experimental, control), face type (critical face, comparison foil face), selection history (selected, not selected), and time bin (250–500 … 1750–2000) was calculated. Proportion of viewing time was well matched across groups (F(1,37) = .05, p = .83) and first-level interactions of face type, selection history, and time bin with group status were not statistically reliable (all F's≤.72, p's≥.43). As reported for global viewing time measures, more time was spent looking at faces that were selected (main effect of selection history: (F(1,37) = 105.86, p < .001). In addition, the distribution of viewing changed over time (main effect of time bin: (F(2.75, 101.88) = 5.11, p = .003; G-G ε = .46) and the effect of selection on eye movement behavior became increasingly robust with time (selection history x time bin interaction: F(2.33, 86.32) = 4.21, p = 0.01; G-G ε = .39). While there was not a main effect of face type (F(1,37) = .11, p = .74), there was a statistically reliable 3-way interaction of face type, time bin, and group assignment (F(3.35, 123.98) = 3.31, p = .02; G-G ε = .56) as well as a statistically reliable 4-way interaction of face type, selection history, time bin, and group assignment (F(4.03, 149.05) = 2.42, p = .05; G-G ε = .67). As can be seen in Fig 4B, these results seem to reflect the influence of memory for scene-face relationships on patterns of viewing among experimental, but not control group participants. The absence of a memory effect among control group participants is not surprising as there was not a studied associate of the subliminal scene cue in the corresponding 3-face display.

Two separate ANOVAs–one that compared viewing behavior directed to faces that were not selected (i.e. non-selected critical faces and non-selected foils), and a second one that compared viewing directed to selected faces (i.e. selected critical faces and selected foils)–were calculated to unpack the 4-way interaction, and to evaluate memory-based viewing effects absent overarching effects of selection history. The 3-way interaction of face type (critical, foil), time bin (250–500 … 1750–2000), and group was statistically reliable when comparisons were based on data from faces that had not been selected (F(4.35,160.84) = 4.24, p = .002, G-G ε = .72); the same interaction for selected faces was not significant (F(3.28, 121.24) = 1.90, p = .13, G-G ε = .55).

To determine when in time memory had an impact on eye movement behavior post-hoc comparisons were performed for each group separately at individual time points starting with the 250–500ms time bin. These comparisons were corrected for the number of time bins that were evaluated (i.e., 7 time bins within 250–2000ms of display onset). Despite the absence of a significant 3-way interaction, initial comparisons examined whether or not memory-based viewing effects distinguished selected critical faces from corresponding foils. In this case, standard memory-based viewing effects were not found; instead, the only statistically reliable outcome was a disproportionate viewing effect greater for selected foils than for selected critical faces from 250–500ms among participants assigned to the experimental group (t(18) = 3.66, Bonferroni corrected p = .01). As predicted, subsequent comparisons performed on data obtained from participants assigned to the experimental group were consistent with memory-based viewing time predictions. More time was spent viewing critical faces that had not been selected than corresponding foils (also not selected) from 1500–2000ms after display onset (t’s(18)≥3.04, Bonferroni corrected p's≤.05; see Fig 4B). This outcome reflects memory for studied scene-face pairs despite the use of a subliminal scene cue, and therefore provides evidence for the implicit expression of eye-movement-based relational memory effects. When the same comparisons were performed for the control group, there were no differences in patterns of viewing directed to critical faces relative to other selected or non-selected foil faces in the 3-face displays (all p's>.19); this outcome was expected, as the studied associate was not present in the implicit test display for control group participants (see Fig 4B). One final analysis, reported in the S1 Supplementary Materials, replicated these outcomes when a direct comparison of critical face viewing (without consideration of corresponding foils) was performed across groups.

#### Explicit Memory for Scene-Face Relationships

**Fig 5:**
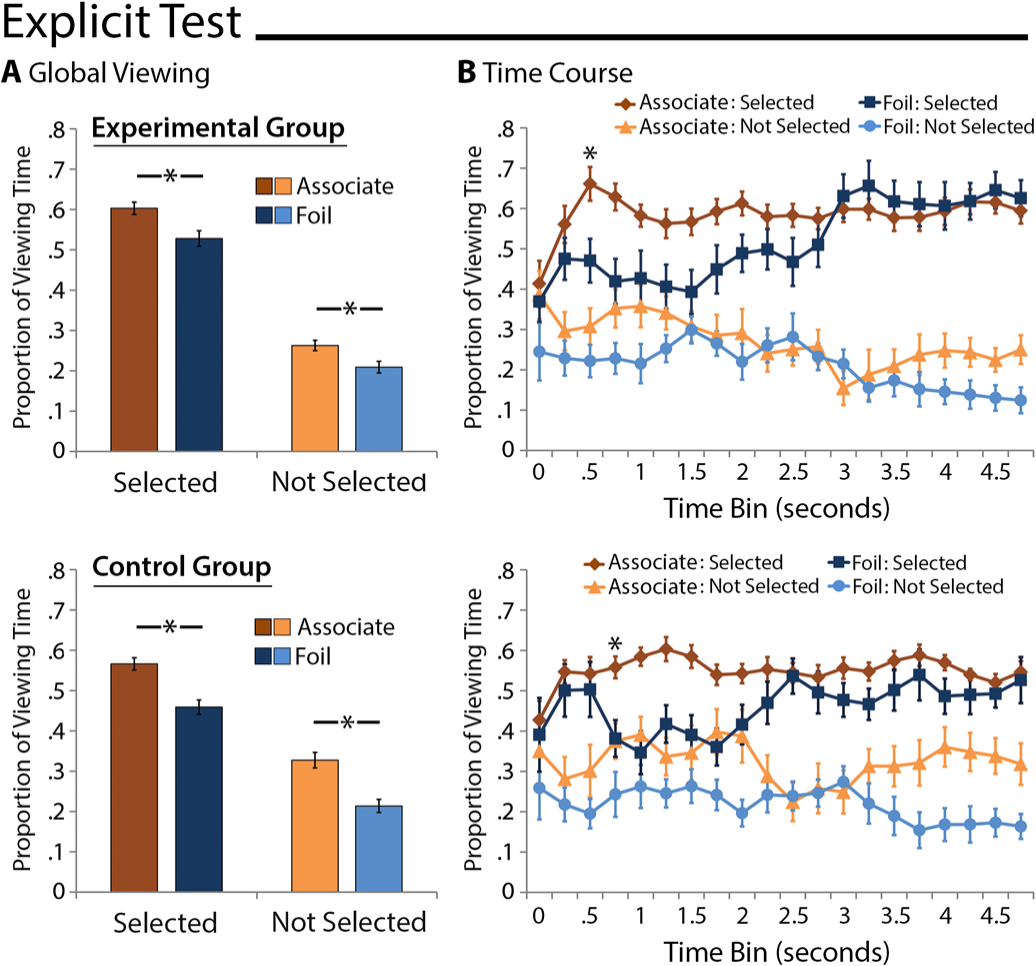
Explicit Memory-Based Viewing Effects. A) Proportion of total viewing time directed to faces of interest collapsed across the duration of the entire test trial for the experimental group (top) and the control group (bottom). More time was spent viewing selected faces, and participants looked disproportionately at the associates of scene cues whether explicit recognition responses were correct or not. B) Proportion of total viewing time directed to faces of interest broken down into 250ms time bins starting with 3-face display onset. Selected faces were prioritized, and memory-based viewing that distinguished correctly identified associates from faces selected in error were evident for both groups shortly after display onset. Error bars indicate standard errors of the mean.

*Global Measures*: Standard memory-based viewing effects, calculated (as for explicit RT analyses) after having eliminated 9 participants for whom there were fewer than 4 incorrect responses, were evident when explicit memory for scene-face pairs was tested. As above, a between-groups repeated measures ANOVA confirmed a main effect of selection history (F(1,28) = 377.22, p < .001)–participants spent more time viewing faces that were selected as compared to those that were not–and this effect was especially robust among experimental group participants (selection history x group interaction: F(1,28) = 8.85, p = .006). Most important for our purposes, and in contrast to implicit memory results reported above, there was also a main effect of face type (F(1,28) = 45.76, p < .001). Follow-up comparisons indicated that participants from both groups spent more time looking at correctly identified associates than at foil faces selected in error (Experimental Group: t(15) = 3.88, p = .001; Control Group: t(13) = 4.07, p = .001). Notably, these memory-based viewing effects were also statistically reliable when comparison were made between associates and foils that had not been selected (Experimental Group: t(15) = 2.70, p = .02; Control Group: t(13) = 3.65, p = .003; see Fig 5A). This outcome indicates that eye movements were sensitive to studied scene-face relationships even when explicit recognition responses were incorrect. Next we examine how soon after display onset these memory-based differences in the distribution of viewing occurred.

*Time-Course Measures*: A between-groups repeated-measures ANOVA with the factors group (experimental, control), face type (associate, comparison foil face), selection history (selected, not selected), and time bin (250–500 … 1750–2000) was calculated. Proportion of viewing time was well matched across groups (F(1,28) = 1.24, p = .28) and first-level interactions of face type, selection history, and time bin with group status were not statistically reliable (all F's≤.1.65, p's≥.18). As reported for global viewing time measures, more time was spent looking at faces that were selected (main effect of selection history: (F(1,28) = 78.34, p < .001) and at studied associates of the visible scene cues (F(1,28) = 47.70, p < .001). This memory-based viewing effect was more robust when associates were correctly identified than they were not (selection history x face type interaction: F(1,28) = 5.72, p = .02), but as can be seen in Fig 5B, differences were in the expected direction whether or not associates were identified and selected. None of the remaining main effects or interactions were statistically reliable (all F’s≤.1.38, p's≥.26).

ANOVAs calculated separately for selected faces (correctly identified associates and foils selected in error) and faces that were not selected (associates that were not identified and foils that were not selected) confirmed outcomes reported above. The only statistically reliable outcome in both cases was a main effect of face type (F’s(1,28)≥21.21, p’s < .001), which was a consequence of more time spent viewing associates than corresponding foils.

Disproportionate viewing of the correctly identified associates relative to faces selected in error was expected to be evident shortly after display onset (i.e., between 500–750ms) for participants assigned to both groups, an effect that would reflect memory for scene-face relationships. Bonferroni corrected post-hoc comparisons indicated that there was significantly more viewing directed to correctly identified associates than to faces selected in error for the experimental group starting at 500–750ms (t(15) = 3.49, Bonferroni corrected p = .02, Cohen’s d = 0.87). The same comparisons made for the control group indicated that disproportionate viewing emerged slightly later in time (750–1000ms time bin: t(13) = 3.46, Bonferroni corrected p = .03; Cohen’s d = 0.92; see Fig 5B). This is notable because there was no a priori reason to expect the temporal onset of this effect to be different across groups–both groups had the same encoding and explicit recognition test experiences. Potential explanations for this unexpected outcome are considered in the discussion section. Evaluation of differences in the amount of viewing time directed to associates that were not correctly identified vs. foils that had not been selected indicated that effects were in the expected direction, but none of the individual time bin contrasts survived correction for multiple comparisons (all t’s≤1.93, Bonferroni corrected p’s≥.51). In sum, these results are largely consistent with past reports, but also go further as most previous studies have not evaluated memory effects when explicit recognition responses were incorrect (i.e. because recognition performance was near ceiling, but see 15).

### Post-Test: Assessment of Awareness

Having identified effects of memory on performance and viewing following the presentation of subliminal scene cues, it is important to rule out the possibility that these effects were a consequence of awareness. Results that address this concern are presented in the sections that follow.

#### Attention Task

Similar to the retrieval phase, compliance with attention task instructions was generally good during the post-test–participants failed to make responses on just 4.40% (SD = 6.26) of the trials. For the remaining trials, successful detection/identification of horizontal or vertical line segments embedded in the subliminal masking sequence was high and well-matched across groups (Experimental Group: 83.30% correct, SD = 20.01; Control Group: 89.58%correct, SD = 11.77; t(37) = 1.02, p = .31; Cohen’s d = .38).

#### Subjective Awareness Assessment: Responses to the Post-Experiment Questionnaire

None of the participants reported having seen anything suspicious in the masking sequence when the first round of questions (e.g., did you notice any regularity in the noise while you were performing the attention task) was administered. Following disclosure about the presence of a scene in the masking sequence, a small subset of the participants indicated that they may have caught glimpses of something in the noise sequence occasionally (Experimental Group = 4 participants; Control Group = 5 participants), but none of them could provide reliable information about scene size, location, or content. Based on these outcomes, it seems reasonable to conclude that participants remained unaware of the presence of a visual stimulus in the masking sequence during the experiment proper.

#### Objective Awareness Assessment: 2-Alternative Forced-Choice Scene Identification

Responses to the questions posed on the awareness questionnaire suggest that participants did not suspect the presence of a scene cue in the subliminal masking sequence. As indicated below, results from the objective test of awareness provide converging evidence for a lack of awareness among experimental group participants.

**Fig 6:**
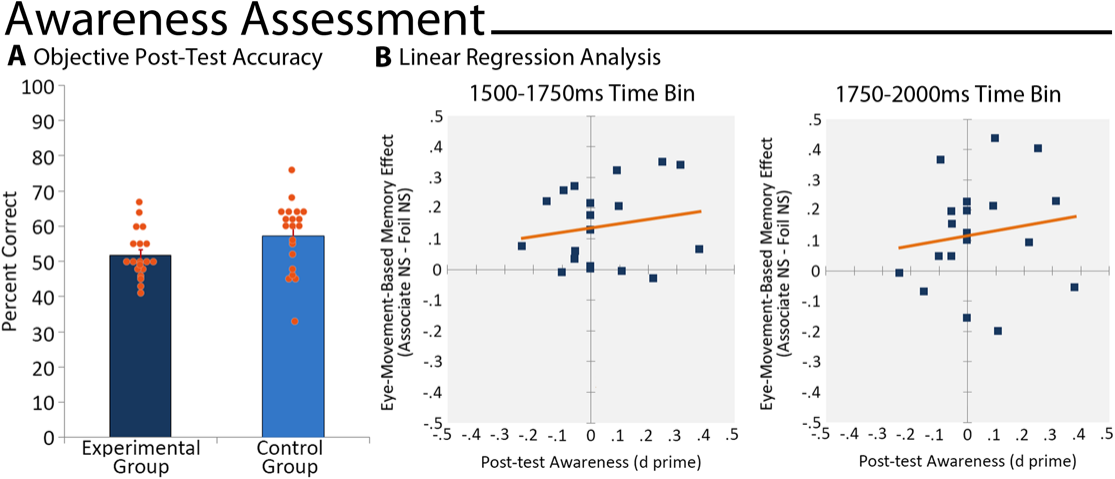
Identification of Subliminal Scene Cues. A) Percentage of scenes that were correctly identified when the objective forced-choice test of scene awareness was adminstered. Results from individual participants are superimposed on the bar graph (orange circles). B) Magnitude of the eye-movment-based memory effects from the 1500–1750ms (left) and 1750–2000ms (right) time bins plotted as a function of post-test awareness (d’ scores). The regression line for each comparison is superimposed.

Collapsed across groups, responses were not made on .55% (SD = 1.39) of the trials. After these trials were eliminated, we found that scene identification was not better than chance for participants assigned to the experimental group (51.80% correct, SD = 6.84; t(18) = 1.15, p = .13); performance was, however, reliably greater than chance for the control group (57.33% correct, SD = 9.89; t(19) = 3.31, p = .002; see Fig 6). Critically, the absence of above-chance performance among participants assigned to the experimental group suggests that memory-based viewing effects on the implicit test were unlikely to have been a consequence of conscious awareness of the masked scene cue. As is evident in Fig 6, however, there was a range of performance across individuals assigned to both groups. Additional analyses, reported in the S1 Supplementary Materials, confirmed that even when participants identified as above-chance performers (via binomial tests) were removed from the data set, effects of memory on eye movement behavior following presentation of subliminal scene cues remained statistically significant.

To determine whether or not recency of the encoding exposure had any effect on visibility of masked scenes presented in the post-test, a between-groups repeated-measures ANOVA with the factor *block* (blocks 1, 2, and 3) was calculated. Consistent with results reported above, controls outperformed participants assigned to the experimental group on the forced-choice identification test (F(1,37) = 4.13, p = .05). However, performance was not influenced by how recently the scenes had been encoded (F(2,74) = .30, p = .75), nor were there differences between groups in performance across blocks (F(2,74) = .22, p = .80). These results confirm that pictures seen recently were not more easily identified than those seen early in the experiment.

#### Linear Regression Analysis

Because a subset of the participants assigned to the experimental group had performances on the awareness test that were numerically greater than chance (see Fig 6), a procedure developed by Greenwald and colleagues [29] was used to determine whether or not implicit, memory-based viewing effects remained statistically reliable when performance on the objective test of awareness was regressed to zero. To perform this analysis, the magnitude of memory-based viewing effects were calculated for each participant, restricted to two time bins of interest when viewing time differences were statistically reliable on the implicit test (i.e. 1500–1750 and 1750–2000ms after display onset). This magnitude score was calculated by subtracting the proportion of viewing time directed to foil faces that had not been selected from the proportion of viewing time directed to corresponding critical faces (i.e. associates of the subliminal scene cue that had not been selected). A score equal to zero would indicate no effect of memory on eye movement behavior. Pearson’s correlations between the magnitude of memory-based viewing effects in each time bin and performance on the objective 2-alternative forced-choice test of awareness (d’ scores) were calculated, and simple regression was used to determine whether or not the implicit viewing time outcome remained statistically significant when post-test awareness was regressed to zero.

Initial tests indicated that there were no statistically reliable correlations between the magnitude of the memory-based viewing effect and performance on the awareness test (1500–1750ms Bin: r(18) = .18, p = .23; 1750–2000ms Bin: r(18) = .15, p = .27). Furthermore, results from the linear regression analysis indicated that successful scene identification on the post-test did not predict the magnitude of implicit eye-movement-based memory effects following subliminal scene cues on the implicit test of memory (1500–1750ms Bin: *slope* = .14, t(18) = .74, p = .47; 1750–2000ms Bin: *slope* = .17, t(18) = .64, p = .53). Finally, results indicated that the y-intercept (i.e. magnitude of viewing) was reliably greater than zero for both time bins (1500–1750ms Bin: y-axis intercept = .14, t(18) = 4.36, p = .001; 1750–2000ms Bin: y-axis intercept = .12, t(18) = 2.76, p = .01), an outcome that indicates memory-based viewing effects were not driven by low levels of awareness among a subset of participants assigned to the experimental group.

## Discussion

The current experiment was designed to address questions about whether or not memory for arbitrary scene-face pairs, shown once each during encoding, can be retrieved following the presentation of a subliminal scene cue. The combined use of subliminal masking procedures and eye movement measures to investigate relational memory retrieval was a departure from past work and meant that we could more effectively address questions about whether memory can be expressed in button-press and/or eye movement behavior despite an absence of awareness. Two primary outcomes suggested not only that subliminal scene cues were processed, but that they triggered pattern completion processes that permit access to representations of learned relationships. First, provided that pairs were successfully encoded, participants assigned to the experimental group selected associates of corresponding subliminal scene cues more often than would be expected by chance. This outcome is considered in light of additional exploratory analyses in the text that follows. Second, following subliminal scene cues, participants assigned to the experimental group spent more time viewing non-selected associates than non-selected comparison faces–an eye-movement-based relational memory effect that is reported here for the first time using subliminal testing procedures. Neither outcome was statistically reliable among controls, which is to be expected, as the studied associates of subliminal scene cues were not present in the corresponding 3-face displays for this group. Consistent with past reports [15] there was also evidence for memory-based viewing time differences when participants made incorrect explicit recognition responses following visible scene cues. Relative to comparison faces, participants from both groups spent more time looking at associates of visible scene cues; this effect was evident even when the associate was not successfully identified as the match. Collectively, these outcomes provide additional converging evidence in favor of the view that eye movements are a sensitive index of memory for studied materials with and without concomitant awareness [12, 14, 24].

Control of scene cue visibility was achieved in this experiment with a masking sequence developed by Henke and colleagues [42]. As in the experiment proper, all of the scenes presented on the objective test of awareness had been seen during the study phase, and results indicated that there were no differences in accuracy as a function of the amount of time between encoding and attempted identification of masked scenes. Critically, mean performance of experimental group participants on this objective forced-choice identification test was not better than chance. However, evaluation of Fig 6 shows that there was a range of performance across participants, which hints at the possibility that memory outcomes on the implicit test were a consequence of scene cue awareness among a small subset of our sample. It is notable that before having been told about the presence of a scene in the masking sequence, none of the participants reported having seen anything unusual or unexpected, but these subjective measures are often described as insufficiently sensitive because participants may adopt an overly strict criterion for reporting awareness [2]. On the other hand, above chance performance on more objective tests (like the one used here) may not index conscious processes selectively (i.e. there may be unconscious contamination) [28]. To address these issues, we used a regression approach advocated by Greenwald and colleagues [29], and found that eye-movement-based memory effects remained statistically reliable when performance on the awareness test was regressed to zero. Furthermore, and as reported in the S1 Supplementary Materials, eye-movement-based implicit relational memory effects remained statistically reliable even when individual above-chance performers were removed from the data set. Collectively then, these outcomes provide strong evidence against the view that conscious identification of the scene cue was driving the reported memory outcomes.

Whether or not other factors might be contributing to reported results was also given due consideration, and indeed, it was discovered that the implicit “memory” effect in back-sorted behavioral data may have been a consequence of selection bias evident in the button-press responses of both groups. That another factor might be contributing to the reported outcome was suggested by control group performance. Controls were marginally more likely to select critical faces from implicit test displays when data were back-sorted as a function of explicit recognition accuracy despite the fact that these faces were not associates of subliminal scene cues. Counterbalancing procedures and follow-up analyses ruled out potential perceptual or location-based influences on reported outcomes, but further analyses did reveal a slight bias among participants assigned to both groups to choose the same face from corresponding implicit and explicit test displays. Of course, it is ideal to make the same response if the face that happened to have been selected was the associate of the visible scene cue presented subsequently, but this bias was evident irrespective of explicit recognition accuracy. It seems then, that when participants were unsure about the identity of the associate, they were sometimes inclined to simply make the same response that had been given previously. It is important to note that this slight response repetition bias had a small impact on explicit recognition memory performance, but that recognition performance was well above-chance for both groups even when all of the repeated responses were eliminated from analyses. Furthermore, the same factor could not have been influencing the reported implicit eye-movement-based memory effect because this data was not back-sorted based on explicit recognition performance, memory-based viewing effects were documented for critical faces that had not been selected (and therefore, were not associated with a button-press response), and controls showed absolutely no preference for critical faces (over foils) in patterns of viewing.

A skeptic might argue that the scene identification approach used to assess awareness in our study was not maximally sensitive, and while that may indeed be the case, we would argue that it was *optimally* sensitive given the objectives of this investigation. When the post-test was administered, participants were instructed to identify the scene that they felt had been presented in the subliminal masking sequence, guessing if necessary. A more conservative test might have used masking sequences that contained embedded scenes on just half of the trials, and required participants to make a presence/absence decision subsequent to each display. Here, we chose not to use the more conservative test because the ability to distinguish scene presence from absence can be supported by low level sensory information (e.g. luminance changes) that is not diagnostic of scene identity. Indeed, if conscious access to low level perceptual factors was sufficient to drive statistically reliable outcomes on the implicit test, then this should also permit successful scene identification on the forced-choice awareness test when participants are deliberately attempting to use any information in the masking sequence to support correct responses [6]. Indeed, it is reasonable to argue that the awareness test was actually too liberal, as it was quite different from implicit test itself. Successful identification of masked scenes on the awareness test could be realized consequent to a simple feature-based matching strategy–participants could compare a line segment, seen in the masking sequence, to the visible scene exemplars and make a reasonable guess about scene identity as a consequence. The same strategy was unlikely to be effective, or even employed, during performance of the implicit test because a simple line segment is unlikely to permit conscious recall of the corresponding scene (from a very large set) and subsequent conscious recovery of information about the associated face. Furthermore, this kind of complex strategy was unlikely to have been used, as participants reported that they were unaware of scene presence on the subjective assessment of awareness. A potential control that could be used in future studies might simply be to change the instruction on the implicit test and ask participants to look at the face they believe was the associate of the subliminally presented scene cue. In this case, participants would know about the presence of the scene, and would attempt to access and use that information to drive viewing behavior. If the reported eye-movement-based memory effects are best characterized as unconscious or unaware, then the pattern observed here should be absent or qualitatively different when participants are given explicit viewing instructions [43].

Finally, it is important to acknowledge that several research investigations indicate that scene gist can be recovered very quickly after stimulus onset [44], although recent studies suggest that early access may be limited to superordinate information (e.g. whether scene content is natural or manmade) [45, 46]. Based on these reports, it is possible that participants would have succeeded on an awareness test at the level of superordinate categorization (e.g. indoor/outdoor discrimination), but again, access to this kind of information is insufficient to support conscious retrieval of specific pairs of studied associates. In sum, questions about whether or not awareness has been completely eliminated are challenging to address, and continue to be a source of considerable debate [3], but here we have demonstrated that even when awareness is minimized, relational memory effects can be documented in eye movement behavior. The methodological approach used here was more conservative than in past studies [20, 24], but the general conclusions remain the same.

While the results described above were generally consistent with our expectations, not all of the predicted outcomes were confirmed, and some of the results were surprising. In contrast to expectations, experimental group participants did not make faster responses when they happened to have selected the associates of subliminal scene cues, and viewing was not directed disproportionately to selected associates following subliminal scene cues relative to other selected faces. Instead, the opposite viewing pattern was observed for a brief time after display onset (i.e. from 250–500ms after display onset). It is possible that this outcome reflects some degree of unconscious uncertainty about the choice that would ultimately be made, but this result should be considered with caution as the group interaction was not statistically significant. In addition, an unexpected, but potentially interesting outcome was a slight difference in the onset of eye-movement-based relational memory effects between groups when memory was tested explicitly. Participants assigned to the experimental group showed standard disproportionate viewing of correctly identified associates that was evident within 500–750ms of display onset [16, 41]. The same difference was not statistically reliable in control data until 750–1000ms, and even then evaluation of Fig 5 suggests that the effect was driven primarily by a decrease in viewing directed to foil faces selected in error, rather than increased prioritization of correctly identified associates. Potential explanations for all of these outcomes are considered in the text that follows.

First, we consider predicted outcomes that were not confirmed. Past studies indicate that subliminal encoding can influence the speed of subsequent decision making when studied materials are presented for conscious inspection [31, 32, 47]. As indicated in the introduction, when participants correctly guess the occupational category of a face following subliminal encoding, they respond more quickly than when their guesses are incorrect. Here, there was no evidence for a difference in the efficiency of responses on the implicit test as a function of whether or not experimental group participants happened to select the associate following subliminal scene cues. While this may seem surprising, response time differences have not always been evaluated in subliminal encoding investigations [35, 36] and in some cases, statistically reliable outcomes consistent with learning have not been observed [42]. It is possible then that the response time effect is subject to specific testing conditions or experimental procedures, which makes it difficult to replicate. In support of this possibility, reported response time differences during test can vary as a function of the speed with which line segments are detected during performance of the attention task (i.e. when subliminal stimulus materials are presented) [47] and as a function of event structure [33]. Additional experiments are needed to identify the factors that determine whether and under what circumstances response time differences occur.

In addition to the lack of response time effects, predicted differences in the amount of viewing time directed to *selected* associates of subliminal scene cues did not materialize. Instead, viewing patterns were dominated by the act of selection itself. Any face that happened to have been selected drew the most viewing, whether that face was the associate of the subliminal scene cue or not. This selection effect was evident shortly after 3-face display onset and remained robust for the duration of the test trial. One potential explanation for this outcome is that the act of selection (required by our instructional manipulation) trumps memory-based viewing of associates that happened to have been selected when participants do not see the scene cues. While similar selection effects were apparent following visible scene cues, there was also clear differentiation between correctly identified associates and faces that were selected in error. This eye-movement-based relational memory effect likely reflects some combination of successful retrieval, which drives viewing to the associate, and uncertainty that accompanies incorrect responses (but see [13]); participants may fixate associates disproportionately when they are retrieved successfully (see [48] for such outcomes with single item test displays) and distribute viewing to other non-selected items in the display (e.g., especially the unidentified associate) when explicit retrieval is not diagnostic. The same kind of deliberation would not be expected following subliminal scene cues when participants are not instructed explicitly to identify the match. More generally, the selection effect that dominates viewing following presentation of subliminal scene cues may have delayed preferential viewing of associates that were not selected (i.e. the subliminal eye-movement-based memory effect described above). Disproportionate viewing of these faces emerged within 1500–1750ms of display presentation, which is 1000ms slower than standard preferential viewing effects (associated with visible scene cues and correct recognition responses) that have been documented in several past studies [15, 16, 41], and are seen here among experimental group participants. Notably though, this delayed time-course is consistent with results of one previous investigation [15]. In that study data were subdivided into 1000ms bins following display onset, and memory-based viewing of associates was evident from 1000–2000ms when explicit recognition responses were incorrect. So, results here provide converging evidence in favor of a slightly delayed time-course of preferential viewing when participants cannot explicitly identify associates of scene cues. In short, when participants remain unaware of scene cues, the immediacy of the selection requirements based on the instructional manipulation may override, curtail, or delay expressions of memory in eye movement behavior, and minimize exploratory search of display elements. This possibility could be evaluated in a future study that eliminates the directive to select a face when 3-face displays follow subliminal scene cues.

One final outcome that is notable concerns a slight difference in the latency of the eye-movement-based relational memory effect between groups on the explicit test. As indicated above, disproportionate viewing of correctly identified associates was delayed among control group participants. It is difficult to draw strong conclusions about this difference in the onset of preferential viewing as there was not a statistically reliable 3-way interaction of group, face type, and time bin; however, we do suggest two potential explanations that could be evaluated in a follow-up investigation provided that the effect can be replicated and a statistically reliable interaction be documented. One possibility is that the difference in time-course is a consequence of priming among experimental group participants, who were exposed to the same scene cue twice, once subliminally and then again supraliminally. However, a priming effect seems unlikely, as disproportionate viewing developed within 500–750ms of 3-face display onset as has been reported in the past [16]. Alternatively, it may be the case that interference between subliminal and visible scene cues, which were different studied scenes for control group participants, slowed allocation of attention and eye movements to correctly identified associates when 3-face displays followed visible scene cues. Similar interference has been reported using response time measures when participants are asked to indicate whether a visible digit, preceded by a masked prime, is less than or greater than 5. When masked primes had semantic identities that were incongruent with the visible target (e.g., a masked 3 followed by a visible 7), response time costs were evident in behavioral responses [6]. This outcome suggests that participants have unconsciously applied the instruction to the prime itself. Consistent with this possibility, the same group has demonstrated, using event-related potentials, that primes were analyzed up to the level of motor response preparation that was consistent with the instructional manipulation [49]. Extending this observation to findings reported here, it is possible that presentation of the subliminal scene cue triggered unconscious retrieval of the associated face, and that this representation interfered with rapid retrieval of the correct associate when visible scene cues were presented subsequently. Again, this is a question that requires further evaluation. One possibility is that the 3-face test display interposed between subliminal and supraliminal scene cues attenuated the interference effect; the time-course difference may therefore be more robust if the initial 3-face display was removed from the trial structure.

Finally, it is worth speculating about the neural correlates of subliminal memory-based viewing effects that were documented in this experiment. It is well established that the hippocampus and adjacent medial temporal lobe cortical structures are critical for long-term declarative (i.e. consciously accessible) memory [50] but there is also growing support for the view that the hippocampus is best characterized by its role in relational memory binding, irrespective of awareness [37, 51]. Indeed, the task used here was adapted from previous studies that have addressed questions about the dependence of eye-movement-based relational memory effects on the hippocampus [15, 16]. These studies have shown that patients with hippocampal damage fail to show any evidence for relational memory in eye movement behavior, even when explicit identification of the matching face happens to have been correct, and that activity differences in the hippocampus predict early disproportionate viewing among neurologically intact individuals, even when explicit recognition responses are incorrect. It seems reasonable, based on these reported outcomes and the proposed role of the hippocampus in relational memory binding and retrieval [52, 53], that the memory-based viewing patterns observed here subsequent to subliminal scene cue presentations are supported by the hippocampus. This could be tested in future work that combines fMRI and eye tracking methods.

In sum, the results reported here provide new insight into the nature and type of representational content that can be recovered and accessed despite an absence of awareness. To date, much of the work that has evaluated unconscious processing has used overlearned materials with which participants have considerable experience, and tasks require semantic decisions (e.g., words, digits [5, 7, 8], but see [54]). Here, we have demonstrated that memory for arbitrarily paired scenes and faces, presented just once during encoding, can be accessed and influence eye movement behavior when scene cues are presented subliminally. That eye movements can be affected by subliminal stimulus exposure has been documented in at least one other investigation. In that experiment, subliminal presentation of objects influenced the efficiency of subsequent change detection performance [55]. Results showed that gaze was attracted more quickly to the location of the change when the subliminal object was the search target than when it was a distractor. This experiment illustrates the utility of gaze analysis in subliminal research studies, but results reported here go further because a direct perceptual match between the subliminal stimulus and items in the test display were not driving reported outcomes. Implicit memory effects were not forthcoming in button-press response data (i.e. selection of the critical face or response time differences). This outcome is consistent with the view that eye movements, which permit moment-to-moment evaluation of data, may represent a more sensitive index of past experience than discrete button-press responses and subjective reports [12]. It is possible that our instructions (i.e. a test of foresight) or the relatively lengthy duration of 3-face displays (i.e. 5 seconds) obviated any implicit memory effects that might otherwise have been observed in the button-press response data–this is something that could be addressed in future research. That said, the eye movement results were robust, stood up to several additional exploratory analyses, and are informative on their own.

## Supporting Information

S1 Data Button-Press and Eye Tracking Data Associated with Implicit/Explicit Test Displays and the Post-Test Assessment of Awareness. (XLSX)

S1 Supplementary Materials Subliminal Norming Procedure and Supplementary Data Analysis. (DOCX)
