# Supplementary material for: Relational Memory Is Evident in Eye Movement Behavior despite the Use of Subliminal Testing Methods
Source: PLoS One. 2015 Oct 29;10(10):e0141677. doi: 10.1371/journal.pone.0141677 (PMC4626025; doi:10.1371/journal.pone.0141677)
Supplement: S1 Supplementary Materials — (DOCX) [file pone.0141677.s002.docx]

**S1 Supplementary Materials**

**Methods**

*Subliminal Scenes: Norming Procedures*

Sixty-seven undergraduate students gave informed consent and participated in a norming task that was used to eliminate visible scene exemplars from the set carried forward for use in the experiment proper. Procedures were approved by the Institutional Review Board at UWM and participants were compensated with course credit. Trial structure and timing parameters matched those used in the awareness test described in the manuscript (see Figure 3). Briefly, on every trial, participants were presented with a scene embedded in a masking sequence and then had to select that scene from two visible alternatives, guessing if necessary. Subsequent to their choice, participants were asked to describe any perceptual details they had noticed when the masking sequence was in view.

The initial round of testing (N=20) was conducted with the entire set of grayscale scenes absent the superimposition of a semitransparent gray layer. In subsequent testing (N=47), increasingly opaque versions of successfully identified pictures were used (see Figure 1) and testing was repeated until a reasonable level of opacity was identified for each scene.

As indicated in the manuscript, the maximum amount of degradation was 70% and more than half of the scenes (n=96) were carried forward without any degradation. Binomial tests, performed for each picture individually, indicated that scene identification was not reliably greater than chance for 145 scenes. A small subset of scenes (n=6) with chance detection rates in the absence of an opacity change were flagged by the experimenters as potentially identifiable, and opacity was increased accordingly. Remaining scenes (n=17) were identified at above-chance levels, but were carried forward for use in the experiment proper based on the absence of any indication in subjective reports that participants saw diagnostic scene information while the masking sequence was in view. The decision to rely on subjective judgments for this small subset of scenes was the result of inconsistent masking success with different levels of opacity (i.e. null identification at lower levels of opacity and above-chance identification at higher levels of opacity). Additional analyses, reported below (see “Excluding Above-Chance Subliminal Scene Identification”), confirmed that the use of these scenes did not drive eye-movement-based memory effects following the presentation of subliminal scene cues.

**Results**

*Potential Contributions of Response Bias to Back-Sorted Implicit Behavioral Results*

As reported in the manuscript, participants assigned to the experimental group selected critical faces (i.e. associates of subliminal scene cues) from 3-face displays at above-chance rates when data were back-sorted based on explicit recognition performance. However, the same effect was marginal among controls, despite the absence of an encoded relationship between subliminal scene cues and critical faces. Because this outcome was unexpected, additional analyses were performed to determine whether or not the implicit memory effect was being influenced/driven by another factor. The appearance or perceptual qualities of critical faces could not account for this result because, across participants, each face was presented more often as a foil than a critical face. Consequently, any preferences for a small subset of faces would make it more difficult to document implicit memory effects.

Another potential contributor to reported outcomes is position bias, a tendency to select faces that occupy particular locations in the 3-face display more often than others. For this to be a viable explanation, critical faces would have to occupy one spatial location more often than the remaining two, and participants would have to show a bias to select faces that occupy that location over the alternatives. To explore this possibility, the percentages of left, right, and bottom critical face appearances across trials included in the back-sorted implicit memory analysis were calculated. For participants assigned to the experimental group, critical faces appeared in the left, right, or bottom positions 33.49, 32.33, and 34.19 percent of the time. For controls, corresponding percentages were 33.94, 32.70, and 33.36. A repeated measures ANOVA with the factors group (control, experimental) and position (left, right, bottom) confirmed that there were no differences in the distribution of critical faces across locations (all F’s≤.15, all p’s≥.55), which meant that any tendency to select faces that occupied one location over the remaining two could not account for the implicit memory effect.

One final source of bias that we considered concerned potential relationships between implicit and explicit responses – specifically, a bias to make the same response to corresponding explicit and implicit test displays. As reported above, results showed that participants from both groups selected critical faces at chance levels (i.e. no more than 33% of the time) when all of the implicit test trials were considered. This means that they should have modified/updated their responses 67% of the time on the explicit test to make a correct identification (keeping the same response just 33% of the time). Evaluation of the data revealed slightly elevated levels of common implicit and explicit test responses for both groups, a bias that was numerically greater among individuals assigned to the experimental group (41.33% retained responses, SD=14.82; control group: 35.62% retained responses, SD=8.65%). The comparison of selection bias across groups was not statistically reliable (t(37)=1.48, p=.15), and selection bias was only greater than chance levels among experimental group participants (t(18)=2.35, p=.02), but the same bias was marginal among controls (t(19)=1.20, p=.12). This pattern of data mimics the pattern reported in the manuscript when implicit responses were back-sorted based on explicit recognition performance and may therefore account for the apparent implicit memory effect.

As might be expected, this choice bias inflated explicit recognition accuracy. Participants from both groups were more likely to make correct explicit recognition responses when critical faces had been selected on the implicit test (Control Group: 84.31% correct, SD=14.43; Experimental Group: 82.48% correct, SD=18.86) than when they were not (Control Group: 78.49% correct, SD=10.35; t(19)=1.76, p=.09; Experimental Group: 68.31% correct, SD=23.11; t(18)=2.78, p=.01). However, the performances of both groups were well above chance (33% correct) irrespective of implicit test responses (t’s≥6.60, p’s<.001). As indicated in the manuscript, we suspect that when participants were unsure about the identity of the associate on the explicit recognition test, they were inclined to keep the same response that had been made previously.

*Group Differences in Critical Face Viewing on the Implicit Test*

In the manuscript comparisons are made between critical faces and corresponding foils. When foils were selected from implicit displays, the remaining two faces in the display (i.e. the associate and the second foil) were also used in reported analyses. Use of all three faces from a given test display (i.e. when a foil has been selected) means that viewing directed to the selected foil detracts from total viewing time available for distribution among the remaining faces (i.e. distribution of viewing to different face types is not independent). Critically though, it is also the case that if memory has no impact on eye movement behavior, residual viewing (regardless of time spent on the selected foil) should be equally distributed among the two other faces in the display (i.e. the associate and the remaining foil); in the absence of a memory-based contribution, there is no reason to prioritize the associate. This was the pattern evident among controls, for whom there was not a match, on the implicit test.

To demonstrate that memory-based viewing is not a consequence of having evaluated eye movements to faces from the same display, an alternative approach is summarized here. Between-group comparisons were calculated based on viewing time directed to just one face per trial – i.e., the critical face. Participants assigned to the control group will show normative patterns of viewing to critical faces, as their appearance in the test display is not preceded by the corresponding scene cue, and there is no opportunity for expression of memory in eye movement behavior. Therefore, if viewing patterns of experimental group participants are not affected by memory for scene-face relationships, there shouldn’t be any between-groups differences. However, if memory does have an effect, we would expect viewing time to be elevated among experimental group participants.

A repeated-measures ANOVA with the factors selection history (selected, not selected), time bin (250-500 … 1750-2000), and group (experimental, control) was calculated after having excluded foil faces from the data. Consistent with implicit time-course results reported in the manuscript, participants spent more time viewing critical faces that had been selected than those that were not selected (F(1,37)=76.71, p<.001), and viewing patterns changed over the course of the trial (F(3.08,114.07)=5.31, p=.002, G-G ε=.51). Most important for our purposes, the 3-way interaction of selection history, time bin, and group was statistically significant (F(2.79,103.07)=3.31, p=.03, G-G ε=.46), which indicates that there were group differences in patterns of viewing directed to critical faces across time.


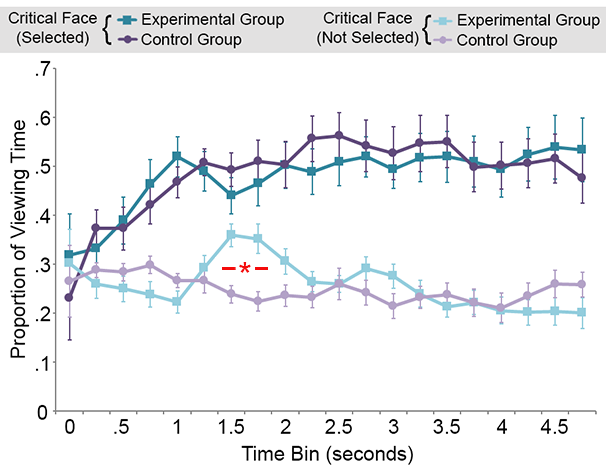
 To unpack this interaction, 2 separate ANOVAs – one for critical faces that had been selected and another for critical faces not selected – were calculated. Results were replicated here as well. When critical faces were selected, patterns of viewing changed with time (F(2.75,101.88)=5.11, p=.003, G-G ε=.46), but patterns of viewing directed to selected critical faces were well matched across groups (F(2.75,101.88)=.72, p=.63, G-G ε=.46). There were, however, statistically reliable differences in time-course of viewing directed to critical faces that had not been selected across groups (F(3.24,119.86)=7.31, p<.001, G-G ε=.54). This differences was a consequence of elevated viewing among experimental group participants to critical faces within 1500-1750ms of 3-face display onset (t’s(37)≥3.68, Bonferroni corrected p’s≤.005); these are the same time bins that were identified in the full analysis (see Supplementary Figure 1). Evaluation of individual time bins revealed no group differences in the amount of viewing time directed to selected critical faces. In short, implicit eye-movement-based relational memory effects were replicated even when statistical contrasts were limited to a single face from each test display.

**Supplementary Fig 1:** Group differences in viewing time to critical faces that were selected and those that were not selected.

*Excluding Above-Chance Subliminal Scene Identification*

Supplementary analyses were also conducted for the implicit test to confirm that statistical outcomes reported in the manuscript were not a consequence of: 1) having used normed scenes with above-chance identification rates, or 2) the above-chance performances of a small subset of participants who completed the experiment proper.

As indicated above, 17 (of 168) scenes carried forward for use in the experiment proper were identified more often than would be expected by chance during norming. To rule out concerns that implicit test results were driven by the inclusion of these scenes, analyses were performed after trials in which they appeared were removed from the data set.

In a separate analysis, individuals who completed the experiment proper, and had above-chance post-test identification rates were eliminated from analyses. This was done to rule out concerns that eye-movement-based memory effects documented subsequent to subliminal scene cue presentation were due to conscious perception of masked scenes among good post-test performers. As a reminder, Experimental Group performance on the post-test (used to evaluate awareness) was not reliably above chance, but data plotted in Figure 6 show that there was a range of performance among individuals assigned to this group. Binomial tests were calculated and participants with above-chance identification rates on the post-test (Experiment Group: N=2; Control Group: N=6) were identified and eliminated from analyses reported below. Subsequent to the elimination of data from these participants, successful identification of masked scenes on the post-test was not better than chance for either group (Experimental Group: 50.19% successful identification, SD=5.13; t(16)=.15, p=.44; Control Group: 53.21% successful identification, SD=8.56, t(13)=1.40, p=.09).

Results based on the evaluation of time-course measures were consistent with those reported based on use of the full data set. More viewing time was directed to faces that had been selected than to those that were not selected (main effect of selection history: F(1,37)=108.05, p<.001 and F(1,29)=78.13, p<.001, excluding above-chance scenes and above-chance participants, respectively), and differences in viewing patterns emerged over the course of the test trial (main effect of time bin: F(2.75,101.63)=3.03, p=.04, G-G ε=.46 and F(2.48,71.84)=3.24, p=.04, GG epsilon=.41, excluding above-chance scenes and above-chance participants, respectively). In contrast to results that were reported based on use of the full data set, the 4-way interactions of selection history, face type, time bin, and group were marginal (F(4.16,153.98)=1.69, p=.15, G-G ε=.69 and F(4.10,118.79)=2.08, p=.09, G-G ε=.68). However, statistical tests performed separately for selected faces (selected targets, selected foils) and faces that were not selected (targets not selected, foils not selected) showed the expected pattern with significant 3-way interactions of face type, time bin, and group when analyses were limited to faces that had not been selected (F(4.38,162.19)=4.52, p=.001, G-G ε=.73 and F(4.26,123.62)=3.73, p=.006, G-G ε=.71, excluding above-chance scenes and above-chance participants, respectively). As reported in the manuscript, 3-way interactions for selected faces were not statistically reliable (F(3.60,133.30)=.89, p=.46, G-G ε=.60 and F(3.25,94.14)=1.74, p=.16, G-G ε=.54, excluding above-chance scenes and above-chance participants, respectively). Post-hoc comparisons confirmed that participants assigned to the Experimental Group directed more viewing time to non-selected targets than to matched foil faces from 1500-2000ms post 3-face display onset (Bonferroni corrected t’s(18) ≥3.39, p’s≤.02 and t’s(16)≥2.92, p’s≤.01, excluding above-chance scenes and above-chance participants, respectively); this is the same memory-based viewing effect that was reported based on analysis of the full data set. The same comparisons, performed with Control Group data, were not statistically reliable (all p’s>.05). As described in the manuscript, the absence of memory-based viewing effects among controls is expected, as none of the faces were the associates of subliminal scene cues for this group.
